# “All-In-One” Genetic Tool Assessing Endometrial Receptivity for Personalized Screening of Female Sex Steroid Hormones

**DOI:** 10.3389/fcell.2021.624053

**Published:** 2021-02-15

**Authors:** Pavel Deryabin, Alisa Domnina, Inga Gorelova, Maxim Rulev, Mariya Petrosyan, Nikolay Nikolsky, Aleksandra Borodkina

**Affiliations:** ^1^Mechanisms of Cellular Senescence Group, Institute of Cytology of the Russian Academy of Sciences, Saint-Petersburg, Russia; ^2^Department of Intracellular Signaling and Transport, Institute of Cytology of the Russian Academy of Sciences, Saint-Petersburg, Russia; ^3^Department of the Assisted Reproductive Technologies, Almazov National Medical Research Centre, Saint-Petersburg, Russia; ^4^Pharmacology Group of D.O. Ott Institute of Obstetrics, Gynecology and Reproductology, Saint-Petersburg, Russia; ^5^The Laboratory of Myocardial Metabolism, Almazov National Medical Research Centre, Saint-Petersburg, Russia

**Keywords:** endometrial stromal cells, decidualization, *in vitro* fertilization, progesterone, transposons, luteal phase

## Abstract

Endometrium is the uterine lining that undergoes hundreds of cycles of proliferation, differentiation, and desquamation throughout a woman's reproductive life. Recently, much attention is paid to the appropriate endometrial functioning, as decreased endometrial receptivity is stated to be one of the concerns heavily influencing successes of embryo implantation rates and the efficacy of *in vitro* fertilization (IVF) treatment. In order to acquire and maintain the desired endometrial receptivity during IVF cycles, luteal phase support by various progestagens or other hormonal combinations is generally recommended. However, today, the selection of the specific hormonal therapy during IVF seems to be empirical, mainly due to a lack of appropriate tools for personalized approach. Here, we designed the genetic tool for patient-specific optimization of hormonal supplementation schemes required for the maintenance of endometrial receptivity during luteal phase. We optimized and characterized *in vitro* endometrial stromal cell (ESC) decidualization model as the adequate physiological reflection of endometrial sensitivity to steroid hormones. Based on the whole transcriptome RNA sequencing and the corresponding bioinformatics, we proposed that activation of the decidual prolactin (PRL) promoter containing ancient transposons MER20 and MER39 may reflect functioning of the core decidual regulatory network. Furthermore, we cloned the sequence of decidual PRL promoter containing MER20 and part of MER39 into the expression vector to estimate the effectiveness of ESC decidual response and verified sensitivity of the designed system. We additionally confirmed specificity of the generated tool using human diploid fibroblasts and adipose-derived human mesenchymal stem cells. Finally, we demonstrated the possibility to apply our tool for personalized hormone screening by comparing the effects of natural progesterone and three synthetic analogs (medroxyprogesterone 17-acetate, 17α-hydroxyprogesterone caproate, dydrogesterone) on decidualization of six ESC lines obtained from patients planning to undergo the IVF procedure. To sum up, we developed the “all-in-one” genetic tool based on the MER20/MER39 expression cassette that provides the ability to predict the most appropriate hormonal cocktail for endometrial receptivity maintenance specifically and safely for the patient, and thus to define the personal treatment strategy prior to the IVF procedure.

## Introduction

Infertility is a global public health issue of modern healthcare that affects a significant proportion of humanity according to the (World Health Organization., 2020). *In vitro* fertilization (IVF) is considered to be the leading approach for infertility curing. Though this type of assisted reproductive technology (ART) seems to be the most effective, in fact, the rate of positive outcomes does not exceed 30% (Gleicher et al., 2019). The “bottleneck” heavily influencing the success of IVF treatment is adequate embryo implantation requiring embryo-endometrial synchronicity (Salker et al., 2010; Teh et al., 2016). To this end, embryo transferring should be performed in the strictly defined time point, when endometrium is characterized by the maximal receptivity to embryonic signals. Importantly, recent observations suggest that endometrial dysfunction (decreased endometrial receptivity) is the cause of implantation failure in about 30% of cases (Tomari et al., 2020).

During each natural cycle, there is only a short period of time termed “window of implantation,” when endometrium transforms into a receptive tissue that enables embryo implantation. The essential step of such transformation is decidualization of the uterine stromal compartment, specifically of the endometrial stromal cells (ESCs) (Okada et al., 2018). Decidualization is initiated during the midluteal phase of the menstrual cycle ~6 days after the ovulation marking the onset of the window of implantation. This tissue-specific differentiation of ESCs is governed by ovarian steroid hormones, particularly by the elevated levels of progesterone, that induce accumulation of the intracellular cyclic adenosine monophosphate (cAMP) and activation of the progesterone and/or cAMP-regulated signaling networks (Okada et al., 2018). Considerable alterations in gene expression profile during ESC decidualization drive dramatic morphological and functional changes, including cytoskeletal and extracellular matrix rearrangements facilitating trophoblast invasion and significant modulation of the secretory activity that contributes to trophoblast growth, prevention of the maternal immunological rejection, and promotion of angiogenesis (Gellersen and Brosens, 2014; Okada et al., 2018). Therefore, decidualized ESC play a crucial role in the establishment of a pregnancy, whereas impairment of this process can lead to a variety of pregnancy complications and might be the cause of implantation failure in IVF (Gellersen and Brosens, 2014; Okada et al., 2018; Deryabin et al., 2020; Tomari et al., 2020).

In order to acquire and maintain the desired endometrial receptivity during IVF cycles, luteal phase support by progestagens is generally recommended (Clinical Practice Guidelines from the Practice Committee of American Society for Reproductive Medicine, 2008; National Collaborating Centre for Women's and Children's Health, 2013). The need for luteal phase support results from exogenous hormonal administration that is commonly used for the artificial regulation of the menstrual cycle during IVF (Palomba et al., 2015). Such hormonal treatment may cause variations in the supraphysiological progesterone and estradiol levels in the early luteal phase leading to asynchrony between the embryo and endometrium, therefore luteal phase support is required. A plenty of meta-analysis performed either for fresh, frozen, or donor embryo transfers confirms that luteal phase support exerts a significant positive effect on clinical pregnancy (Van der Linden et al., 2011; Palomba et al., 2015). Although scientific society agreed to recommend exogenous progesterone for luteal phase support, a lot of clinical trials aimed to test its synthetic analogs and various hormonal combinations to find a “golden standard” for increasing IVF effectiveness (Abate et al., 1999; Yu et al., 2018; Brum Scheffer et al., 2019; Fusi et al., 2019; Griesinger et al., 2019). However, search for the universal approach might not be entirely a rational strategy, as endometrium of different patients can vary significantly in the degree of maturation, expression of progesterone and estrogen receptors, and other marker molecules (Díaz-Gimeno et al., 2011). Moreover, female infertility may be accompanied by various gynecological complications, e.g., tubal obstruction, anovulation, infection, polycystic ovarian syndrome, premature ovarian failure, and so on (Vannuccini et al., 2016). These observations testify that the exact hormonal supplementation scheme should rather be patient specific than universal to achieve more efficacies in IVF. Mainly due to the lack of the appropriate tools for personalized approach, today the selection of the concrete hormonal therapy during IVF seems to be empirical.

The aim of the present study was to develop easy-to-use and cost-effective tool for personalized screening of female sex steroid hormones based on the endometrial receptivity assessment. The design of this instrument included several stages. Firstly, we optimized and characterized *in vitro* ESC decidualization model as the adequate physiological reflection of endometrial sensitivity to steroid hormones. Secondly, based on the RNA-seq and bioinformatic analyses, we proposed that activation of the decidual PRL promoter containing ancient transposons may serve as an integral index of the decidual regulatory network functioning. Thirdly, using the sequence of decidual PRL promoter containing MER20 and part of MER39, we constructed expression vector to estimate the effectiveness of ESC decidual response and verified sensitivity and specificity of the designed system. Finally, we confirmed the possibility to apply our tool for personalized hormone screening by comparing the effects of natural progesterone and three synthetic analogs on decidualization of six ESC lines obtained from patients planning to undergo the IVF procedure.

## Materials and Methods

### Cell Cultures

Patients' samples of menstrual blood containing fragments of the desquamated endometrium were obtained under a cooperation agreement with the Almazov National Medical Research Center. Human ESCs were isolated from desquamated endometrium according to the procedure described previously (Zemelko et al., 2012). The study was reviewed and approved by the Local Bioethics Committee of the Institute of Cytology of the Russian Academy. Human diploid fibroblasts, adipose-derived human mesenchymal stem cells and HEK293T cells were obtained from Russian Cell Culture Collection (Institite of Cytology RAS, Saint-Petersburg). All cells were cultured in DMEM/F12 (Gibco BRL, USA), except for HEK293T that were cultured in DMEM (Biolot, Russian Federation) at 37°C in a humidified incubator, containing 5% CO_2_. Cultural media was supplemented with 10% FBS (HyClone, USA), 1% penicillin-streptomycin (Gibco BRL, USA), and 1% GlutaMAX (Gibco BRL, USA). Serial passaging was performed when the cells reached 80–90% confluence. For the experiments, cells at early passages were used.

### Decidualization Induction

After the cells reached 80% density, the medium was exchanged for serum-free medium for 24 h. The next day, medium was replaced by the fresh medium containing 2% of serum and 0.3 mM N6,2′-*O*-dibutyryladenosine 3′,5′-cAMP (Sigma-Aldrich, USA), 10 nM β-estradiol (E2) (Sigma-Aldrich, USA), and 1 μM medroxyprogesterone 17-acetate (MPA) (Sigma-Aldrich, USA), such medium was exchanged every second day. To compare progesterone and various progestins, MPA in the induction media was replaced by the equivalent amounts of progesterone (Sigma-Aldrich, USA)/17α-hydroxyprogesterone caproate (Merck, USA)/dydrogesterone (Merck, USA).

### Western Blotting

Western blotting was performed as described previously (Borodkina et al., 2014). SDS-PAGE electrophoresis, transfer to nitrocellulose membrane, and immunoblotting with ECL (Thermo Scientific, USA) detection were performed according to standard manufacturer's protocols (Bio-Rad Laboratories, USA). Antibodies against the following proteins were used: glyceraldehyde-3-phosphate dehydrogenase (GAPDH) (clone 14C10) (#2118, Cell Signaling, USA), E-cadherin (clone HECD-1) (ab1416, Abcam, UK), vimentin (clone RV202) (ab8978, Abcam, UK), progesterone receptor A/B (clone D8Q2J) (#8757, Cell Signaling, USA), estrogen receptor α (clone D6R2W), as well as horseradish peroxidase-conjugated goat anti-rabbit IgG (GAR-HRP, Cell Signaling, USA) and antimouse IgG (GAM-HRP, Cell Signaling, USA).

### RNA Extraction, Reverse Transcription, and Real-Time PCR

RNA extraction, reverse transcription, and real-time PCR were performed as described in our previous study (Griukova et al., 2019). Primer sequences and the corresponding annealing temperatures are listed in Table 1.

**Table 1:** Primer oligonucleotide sequences.

***N***	**Oligonucleotide**	**Sequence**	**Annealing temperature**
1	GAPDH forward	5′-GAGGTCAATGAAGGGGTCAT-3′	56.0
2	GAPDH reverse	5′-AGTCAACGGATTTGGTCGTA-3′	56.0
3	ACTA1 forward	5′-AATGCAGAAGGAGATCACGG-3′	57.5
4	ACTA1 reverse	5′-TCCAGACAGAGTATTTGCGC-3′	57.5
5	VIM forward	5′-TATGAAGGAGGAAATGGCTCG-3′	57.5
6	VIM reverse	5′-CCTGTAGGTGGCAATCTCAAT-3′	57.5
7	CDH2 forward	5′-ACCAAAGTCACGCTGAATACA-3′	57.5
8	CDH2 reverse	5′-ACCCAGTCTCTCTTCTGTCTT-3′	57.5
9	TWIST1 forward	5′-GTCCGCAGTCTTACGAGGAG-3′	58.5
10	TWIST1 reverse	5′-GAATCTTGCTCAGCTTGTCCG-3′	58.5
11	FOXO1 forward	5′-TCTACGAGTGGATGGTCAAGA-3′	57.5
12	FOXO1 reverse	5′-ATGAACTTGCTGTGTAGGGAC-3′	57.5
13	IGFBP1 forward	5′-GCAGACAGTGTGAGACATCC-3′	57.5
14	IGFBP1 reverse	5′-GAGACCCAGGGATCCTCTTC-3′	57.5
15	PRL forward	5′-ATGAAGAGTCTCGCCTTTCT-3′	56.0
16	PRL reverse	5′-TGTTGTTGTGGATGATTCGG-3′	56.0
17	CLU forward	5′-AAGAAAGAGGATGCCCTAAATGAG-3′	57.5
18	CLU reverse	5′-TTCATGCAGGTCTGTTTCAGG-3′	57.5
19	ALB forward	5′-TTTGCAGATGTCAGTGAAAGAGA-3′	58
20	ALB reverse	5′-TGGGGAGGCTATAGAAAATAAGG-3′	58
21	WPRE forward	5′-GTCCTTTCCATGGCTGCTC-3′	58
22	WPRE reverse	5′-CCGAAGGGACGTAGCAGA-3′	58
23	CEBP/B forward	CTGTGACCCTGAAGCACCAA	57.5
24	CEBP/B reverse	TTCTTGGCCCACTTCATCCC	57.5
25	PGR forward	CCGCGCTCTACCCTGCAC	57.5
26	PGR reverse	GGGCTCTGGCTGGCTTCTG	57.5
27	ESR forward	CAGGCTTTGTGGATTTGACC	57.5
28	ESR reverse	TCCAAGAGCAAGTTAGGAGC	57.5
29	XBP1 forward	TGAAAAACAGAGTAGCAGCTCAGA	57.5
30	XBP1 reverse	CCCAAGCGCTGTCTTAACTC	57.5

### F-Actin Cytoskeleton Visualization

Cells grown on coverslips were fixed with 4% formaldehyde (15 min), permeabilized with 0.1% Triton X-100 (10 min) and blocked with 1% BSA (1 h). Cells were incubated with rhodamine phalloidin (Thermo Scientific, USA) for 30 min at 37°C and then washed three times with PBS/0.1% Tween 20. The slides were counterstained with 1 μg/ml DAPI (Sigma-Aldrich, USA) and mounted using 2% propyl gallate. A ZOE Fluorescent Cell Imager (BioRad, USA) was used to view and acquire images.

### ELISA

The amounts of secreted PRL and IGFBP-1 were quantified in the cell supernatants by the Prolactine Human ELISA Kit (Abcam, USA) and Human IGFBP-1 ELISA Kit (Sigma-Aldrich, USA). The data were normalized to the total amount of protein determined by the Bradford method. Positive and negative controls provided by the manufacturer were performed in parallel for comparisons. To determine the concentration of secreted proteins in samples, GraphPad Prism 5 was used.

### RNA Preparation and Whole Transcriptome RNA Sequencing

Total RNA was extracted from the non-differentiated and decidualized ESCs using ExtractRNA reagent (Evrogen, Russia) according to the manufacturer's protocol. The concentration of RNA was calculated using the Qubit 3.0 Fluorometer (Thermo Scientific, USA). The quality control was performed with Bioanalyzer 2100 (Agilent, USA) capillary gel electrophoresis. The lower threshold for RIS quality control of the samples was no <9. CDNA was synthesized from total RNA using Mint-2 kit (Evrogen, Russia) according to the manufacturer's protocol. The RNA libraries were prepared with the Qiaseq FX DNA Library kit (Qiagen, Germany). The whole transcriptome RNA sequencing (RNA-Seq) was performed with the HiSeq 2500 sequencing platform (Illumina, San Diego, CA, USA) in the single-end mode and with a read length of 50 bp in a rapid run mode.

### RNA-Seq Read Processing, Transcripts Quantification, and Differential Expression Analysis

Raw data comprising four biological replicates for each condition (non-differentiated and differentiated) were processed as follows. Raw reads underwent quality filtering *via* the FilterByTile script from the BBtools package using the default options (version 38.75) (Bushnell, 2014). The remaining reads were additionally filtered and trimmed with the use of trimFilter script from the FastqPuri package (version 1.0.7) (Pérez-Rubio et al., 2019). In particular, trimming operation was applied for both ends of reads if they contained Ns or their quality was below the quality threshold set to 27, all reads shorter than 25 bases were discarded. The quality control of trimming was held with the FastQC software (version 0.11.7) and FastqPuri scripts (Andrews, 2010). The reads, having passed both operations, comprised no <90% of the initial data, with the average read length close to 50 bases.

For transcript abundances, estimating the salmon lightweight mapping was applied (version 1.1.0) (Patro et al., 2017). The mapping was performed in the selective alignment mode. The list of decoys was generated based on the Gencode human reference genome GRCh38.p13 (release 33) and used further for building the index on concatenated transcriptome and genome Gencode reference files (release 33) using k-mer size of 21. Mapping operations were run with additional flags –numBootstraps 30 –seqBias –gcBias –validateMappings. Resulting mapping rates were around 70%.

**Figure 1:**
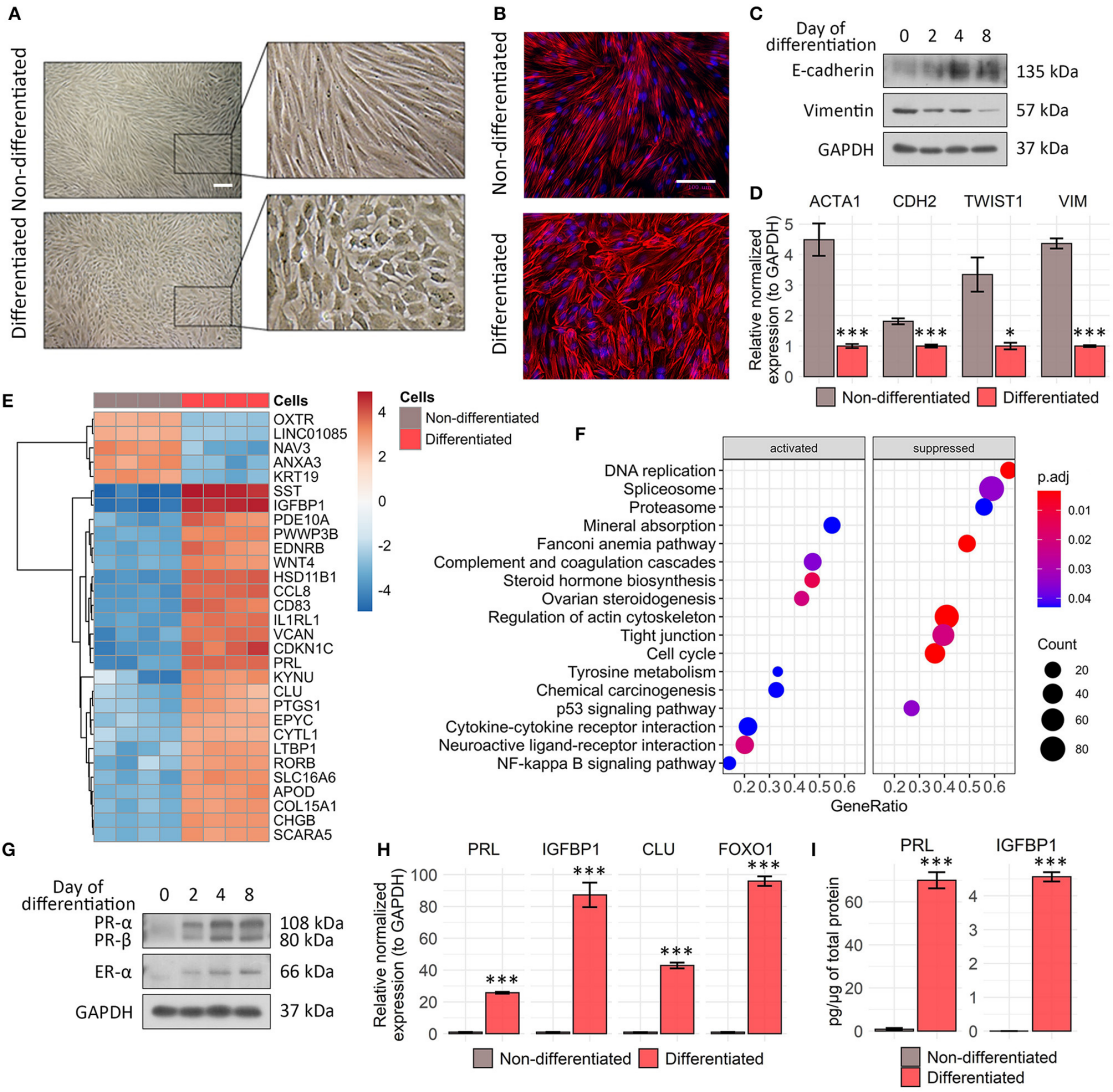
Characteristic features acquired during tissue-specific ESC decidualization. **(A)** ESCs switch morphology from fibroblast like to epithelial like in 8 days after decidualization induction. Scale bar is 500 μm for all images. **(B)** Rhodamine phalloidin staining reflects actin cytoskeleton rearrangements in decidualized ESCs. Scale bar is 100 μm for all images. **(C)** Decidual transformation of ESCs is accompanied by the gradual increase in E-cadherin expression and decline in vimentin expression as indicated by Western blotting with specific antibodies. Representative blots of the three experiments are shown here. GAPDH was used as loading control. **(D)** *ACTA1, CDH1, TWIST*, and *VIM* genes expression levels in ESCs decidualized for 8 days estimated by RT-PCR. Values are M ± S.D. (*N* = 3). *p < 0.05; ***p < 0.005 differentiated vs. non-differentiated cells by Student's *t*-test. **(E)** Heatmap demonstrating top 30 differentially expressed genes in the untreated and decidualized ESCs scaled by normalized and rlog-transformed transcripts counts. **(F)** Gene set functional enrichment analysis of up- and downregulated cellular processes and pathways in differentiating ESCs in KEGG DB terms. **(G)** Western blot analysis of progesterone receptor A/B and estrogen receptor α expression performed during ESC decidualization. Representative results of the three experiments are shown here. GAPDH was used as loading control. **(H)** Analysis of *FOXO1, IGFBP1, PRL*, and *CLU* expression levels by RT-PCR. Values are M ± S.D. (*N* = 3). ***p < 0.005 differentiated vs. non-differentiated cells by Student's *t*-test. **(I)** The amounts of secreted PRL and IGFBP-1 were estimated by ELISA in the cell supernatants of undifferentiated and decidualized ESCs. ELISA values presented as M ± S.D. (*N* = 4). ***p < 0.005 by Student's *t*-test.

Further data processing was performed using R version 3.6.3 with the Tidyverse collection of packages (version 1.3.0). Estimated gene counts, metadata, and transcript ranges were loaded into R using tximeta (version 1.4.5) and summarized to a gene level (Love et al., 2020). Resulting count matrix was filtered to contain rows having at least 10 estimated counts across all samples; the resulting matrix contained 20,428 genes. Gene differential expression (DE) analysis and log fold change (LFC) estimation (Figure 1E, Supplementary Tables 1, 2) were computed using DESeq2 (version 1.26.0) with a design formula controlling for cell differentiation status (Love et al., 2014). To strengthen DE analysis, here, we correct LFC using combination of adaptive shrinkage estimator from the apeglm package (version 1.8.0) and specifying additional LFC threshold equal to 0.667 (Zhu et al., 2018). This testing produced alternative *p*-values or *s* values telling whether the LFC is greater in absolute value than the threshold. Thus, returned *s* values provided the probability of “false signs or small” events (FSOS) among the tests with equal or smaller *s* value than a given genes *s* value, where “small” was specified by LFC threshold (Stephens, 2016). Genes that had *s* value smaller than 0.005 were defined as differentially expressed (LFC >0.67 (up): 1,385, LFC ≤0.67 (down): 1,505).

### Bioinformatic Data Analysis

Heatmaps were constructed with the use of genefilter (version 1.38.0) and pheatmap (version 1.0.12) R packages. Gene set enrichment analysis was conducted using clusterProfiler (version 3.14.3) and fgsea (version 1.12.0) R packages based on ranking genes by estimated shrunken LFC values, and *p*-values were adjusted according to the Benjamin–Hochberg multiple test adjustment and *q*-value cutoff of 0.1 (Yu et al., 2012). Testing gene list in terms of biological processes and pathways was conducted based on KEGG Pathway DB (Kanehisa et al., 2017).

For prediction of transcription factors (TFs) binding to decidual PRL promoter sequence HumanTFDB (AnimalTFDB release 3) and PROMO (version 3.0.2), web applications were used with the confidence thresholds *p* < 0.0001, *q* < 0.1, and dissimilarity <10%, respectively (Farré et al., 2003; Hu et al., 2019). Obtained lists of binding sites were additionally manually filtered from redundancy. Gene set enrichment analysis in terms of regulation by TFs were performed *via* clusterProfiler and fgsea R packges as described above based on TF regulons gathered from the TRED DB (Jiang et al., 2007).

### Dec_pPRL-Mcherry Plasmid Construction

Genomic DNA was isolated using NucleoSpin tissue kit (Macherey-Nagel, Germany) according to the manufacturer's instructions. The DNA fragment −524/+65 around decidual PRL transcription start site was amplified by PCR with primers forward (5′-ACTTTAATTAAGACAGTCTCATCTCCATTATTGACTGCA-3′) and reverse (5′-TTGACCGGTGTCTCTGTCTTTGAGGGTACTTCTG-3′). Amplified PCR product was purified using QIAquick gel extraction kit (Qiagen, Germany). Backbone vector pUltra-hot (https://www.addgene.org/24130/) and the insert were double digested with the following restriction enzymes *Age*I (NEB, UK) and *Pac*I (NEB, UK) at 37°C for 1 h in NEBuffer 1.1 (NEB, UK). Ligation was performed using Quick Ligation kit (NEB, UK). The obtained plasmid was then amplified in Stbl3 chemically competent *Escherichia coli*.

### Lentivirus Production, Titration, and Cell Transduction

Protocols of lentiviral particle production and ESC lentiviral transduction are described in detail in our previous article (Deryabin et al., 2019). For virus titration, ESCs were used. The cells were plated at 10^5^ in 35 mm dishes in 1 ml of the complete medium and the next day were transduced with serially diluted viral stocks in fresh complete medium supplemented with 20 mg/ml of protamine sulfate (Ps) (Sigma-Aldrich, USA) for 18 h. Then the medium was replaced to the “induction” medium containing cAMP, MPA, and E2. Cells were cultured for 8 days and then mCherry fluorescence was analyzed by flow cytometry. Viral integration frequency was assessed according to the procedure described by Barczak et al. (2015). Primers' sequences are presented in Table 1.

### Flow Cytometry Analysis

Measurement of mCherry fluorescence was carried out by flow cytometry using the CytoFLEX (Beckman Coulter, USA) with the peak excitation wavelength for mCherry 587 nm and emission 610 nm. The obtained data were analyzed using CytExpert software version 1.2. At least 10^4^ cells were measured per sample. In order to access cell viability, DAPI (Sigma) was added to each sample just before analysis.

### Statistical Analysis

Unless otherwise indicated, all quantitative data are shown as M ± S.D. To get significance in the difference between two groups, Student's *t*-test was applied. For multiple comparisons between groups, ANOVA with Tukey HSD was used. Statistical analysis was performed using R software.

## Results

### Phenotypic and Transcriptomic Alterations Accompanying *in vitro* Decidualization of Human Endometrial Stromal Cells

In order to develop the appropriate genetic tool to assess endometrial sensitivity to sex steroid hormones, we first optimized *in vitro* model that adequately reproduces ESC decidualization. To this end, we applied the “induction cocktail” containing cAMP, 17-beta-E2, and MPA. To begin with, we analyzed the morphological alterations accompanying decidualization. As shown in Figures 1A, B, ESCs switched morphology from fibroblast like to polygonal epithelial like upon decidualization induction. The revealed morphological shift coincided with the gradual decrease in vimentin expression and simultaneous rise in E-cadherin protein level, suggesting in favor of mesenshymal-to-epithelial transition (MET) (Figure 1C). As the additional confirmation of MET progression during ESC decidualization, we observed the decline in expression levels of *ACTA1, CDH1, TWIST*, and *VIM* genes (Figure 1D).

To further characterize the relevance of our *in vitro* decidualization model, we compared transcriptomic signatures of undifferentiated and decidualized ESCs using RNA-seq. Among 2,890 differentially expressed genes (DEGs) in decidualized ESCs (FSOS *s* value <0.005), 1,385 were upregulated, while 1,505 were downregulated (full list of identified genes is presented in Supplementary Table 1; full list of DEGs is presented in Supplementary Table 2). As expected, all the key participants of the classical decidual response, such as *IGFBP1, PRL, PROK1, FOXO1, PGR, CEBPB, WNT4, LEFTY2*, and *CLU* were upregulated in decidualized ESCs (Figure 1E, Supplementary Table 2). We annotated all the identified genes by KEGG pathway terms to identify molecular pathways that were recruited into ESC decidualization (Supplementary Table 3). We found that several pathways with important roles in decidualization were over-represented among the recruited genes, including “steroid hormone biosynthesis,” “cytokine-cytokine receptor interaction,” and “ovarian steroidogenesis” (Figure 1F). At the same time “cell cycle,” “DNA replication,” and “regulation of actin cytoskeleton” pathways were significantly downregulated upon ESC decidualization (Figure 1F).

To validate the results of RNA-seq, we analyzed protein and gene expression levels of the most important players mediating decidual reaction. Namely, we detected increase in the levels of progesterone (PR) and estrogene receptors (ER) during ESC decidualization (Figure 1G). Both nuclear receptors act as transcription factors upon binding to the corresponding hormones and thus directly regulate expression of a large number of decidual genes. In addition, we observed enhanced expression of *FOXO1*, another core decidual transcription factor (Figure 1H). The above transcription factors are known to be responsible for the expression of the decidual marker genes—*PRL* and *IGFBP1*. Indeed, we revealed significant increase in mRNA levels both of *PRL* and *IGFBP1* (Figure 1H). The relevant proteins encoded by these genes are secreted by decidualized ESCs and serve as biochemical markers for stromal cell differentiation (Okada et al., 2018). Using enzyme-linked immunosorbent assay, we detected substantial elevation in the IGFBP1 and PRL protein contents in the conditioned media collected from decidualized ESCs (Figure 1I).

Together, the results described within this part are mostly in line with the existing ones and clearly demonstrate that the selected treatment design of ESCs sufficiently reflects stromal decidualization (Rytkönen et al., 2019).

### Activation of Decidual *PRL* Promoter Containing Ancient Transposons Reflects Functioning of the Decidual Regulatory Network

**Figure 2:**
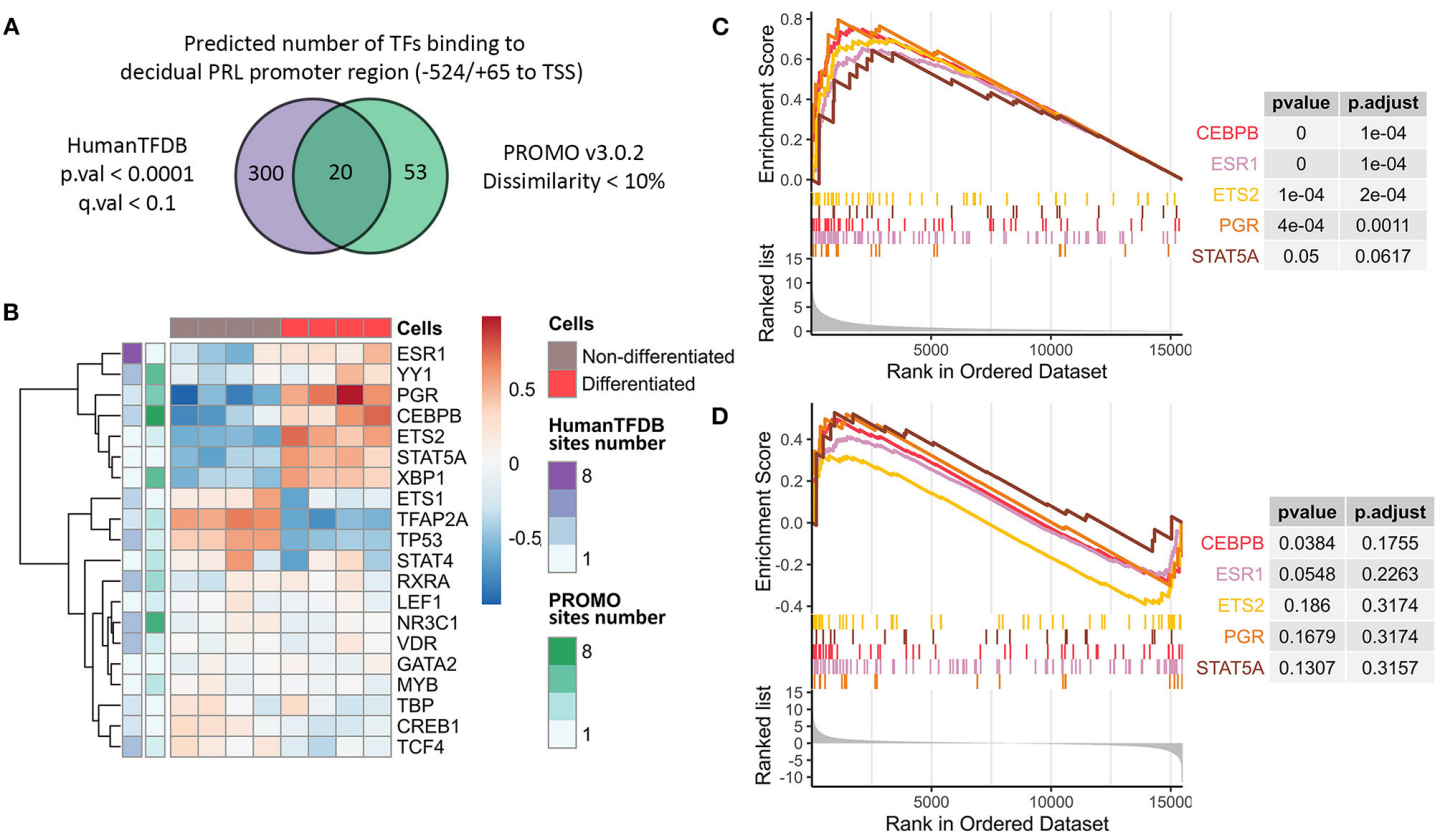
Characterization of decidual PRL promoter as the “core” element for the integral decidualization assessment. **(A)** Venn diagram reflecting the number of predicted TFs able to bind the decidual PRL promoter sequence. **(B)** Expression levels and the numbers of predicted binding sites for the TFs proposed to bind the decidual PRL promoter sequence in decidualized and non-differentiated ESCs. **(C**, **D)** Gene set enrichment analysis in terms of regulation by TFs based on TRED DB built on the pre-ranked gene list by either absolute or original value of LFC from DE analysis of decidualized vs. non-differentiated ESCs, respectively.

Having established the optimal *in vitro* conditions to induce ESC decidualization, we next tried to elucidate the most universal genetic marker that would allow assessing the effectiveness of this reaction, and thus can form the core of the genetic tool for endometrial receptivity estimation. In this context, we paid particular attention to the decidual *PRL* (*dPRL*) promoter as the possible transgene candidate. This promoter controls tissue-specific expression of the decidual marker gene *PRL* in endometrium. *DPRL* promoter localizes 6 kb upstream of the first exon transcribed in the pituitary, therefore, decidual *PRL* transcript contains additional 5′-untranslated exon compared with the *PRL* expressed in the pituitary (Gellersen et al., 1989; Telgmann et al., 1997). The major activity of the *dPRL* promoter is fully covered by the region within −332/+65 from decidual transcription start site (TSS) (Telgmann et al., 1997). This region includes the full-length sequence of MER20 and part of MER39 transposons (Gellersen and Brosens, 2014). *Cis*-regulatory (promoter/enhancer) function of MER20/MER39 region is predominantly mediated by the TF binding sites present within this sequence (Lynch et al., 2011; Emera and Wagner, 2012). By applying two different bioinformatic tools HumanTFDB and Promo v3.0.2, we identified 20 TFs predicted to bind with the *dPRL* promoter sequence with high probability, including CEBP/B, PR, STAT5A, XBP-1, and ER important for pregnancy (Figure 2A). As expected, among the predicted TFs, there were TFs previously proved to bind this region using chromatin immunoprecipitation with quantitative PCR (Lynch et al., 2011).

To drive gene expression, availability and accessibility of binding sites should be essentially accompanied by the appropriate TF repertoire to utilize these binding sites. Importantly, we observed upregulation of almost all the above mentioned TFs in decidualized ESCs, what favors the acquisition of the appropriate TF profile during ESC decidualization (Figure 2B). These results were additionally verified by qPCR (Supplementary Figure 1B). Furthermore, these TFs were predicted to have several binding sites within the analyzed region (Figure 2B).

We then tested whether the revealed TFs are indeed responsible for the regulation of the effector decidual program. To this end, we annotated genes expressed in ESCs in TF regulatory terms (TRED DB) using gene set enrichment analysis. It should be emphasized that it was DEGs that turned out to be regulated by the identified TFs in decidualized ESCs (Figure 2C, Supplementary Table 4). Moreover, we revealed the tendency for the upregulated genes in decidualized ESCs to be predominantly controlled by the defined list of TFs (Figure 2D, Supplementary Table 5).

Thus, insertion of the cassette carrying these transposons may function as a platform for the key transcription factors that regulate ESC decidualization, and its activation may reflect the adequacy of the transcription regulator profile that manages the expression of the whole decidual network in ESCs. Being rather small and at the same time extremely capacious, MER20/MER39 sequence includes a plenty of binding sites for the core TFs that regulate the whole decidual network, what makes it an ideal candidate to estimate the overall decidual response.

### Development and Characterization of the Fluorescent Reporter System Based on the *dPRL* Promoter Activation

**Figure 3:**
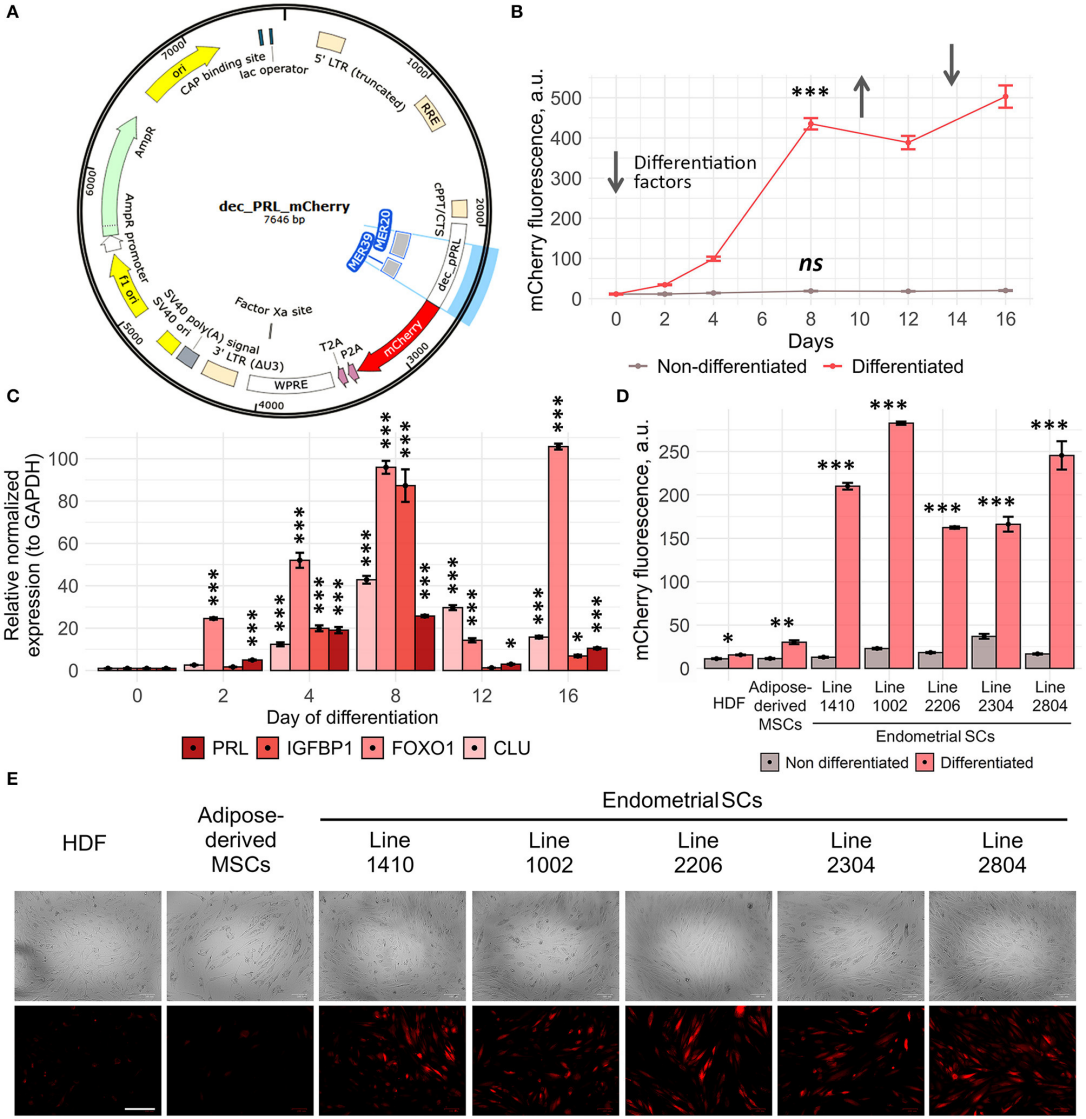
Dec_pPRL-mCherry reporter system allows estimating ESC decidualization. **(A)** Schematic presentation of the transgene cassette containing MER20 and part of MER39 with the corresponding TF binding sites and the designed dec_pPRL-mCherry expression vector. **(B)** Dynamics of ESC decidualization assessed by mCherry fluorescence intensity upon serial addition and removal of the “induction” media. Values are M ± S.D. (*N* = 4). ns, not significant; ***p < 0.005 by one-way ANOVA in differentiated and non-differentiated cell groups. A.u., arbitrary units. **(C)** Modulations of mCherry fluorescence correlated with the alterations in the expression levels of the key decidual genes in ESCs analyzed by RT-PCR, when reproducing the same experimental conditions as in **(B)**. Values are M ± S.D. (*N* = 3). *p < 0.05; ***p < 0.005 by ANOVA with Tukey HSD vs. non-differentiated cells (day 0). **(D**, **E)** Specificity of the developed tool analyzed by the level of mCherry fluorescence in HDF, AdMSCs, and five ESC lines in 8 days after hormonal supplementation either by FACS or by ZOE fluorescent cell imager, respectively. Values are M ± S.D. (*N* = 3). *p < 0.05; **p < 0.01; ***p < 0.005 differentiated vs. non-differentiated cells by Student's *t*-test. A.u., arbitrary units. Scale bars of all images are 200 μm.

According to the results described above, *dPRL* promoter seemed to be a good candidate to become a core fragment of the “all-in-one” genetic tool to estimate integral endometrial receptivity. To this end, we amplified the region −524/+65 around *dPRL* TSS that covers −332/+65 region previously described to be responsible for the full activity of the dPRL promoter (Telgmann et al., 1997). The amplified region was further cloned into the pUltra-hot lentivector (https://www.addgene.org/24130/) instead of UbC promoter, so that mCherry fluorescent protein expression was under control of the *dPRL* promoter (dec_pPRL-mCherry) (Figure 3A). Thereby, the designed genetic construct allows assessing the effectiveness of decidualization simply by mCherry fluorescence intensity.

In order to characterize dec_pPRL-mCherry tool, we performed a series of experiments aimed to estimate its sensitivity and specificity. Firstly, we analyzed the dynamic changes of mCherry fluorescence in transduced ESCs upon addition and removal of the differentiation “induction cocktail.” As shown in Figure 3B, mCherry fluorescence gradually increased during 8 days of the constant presence of the “induction cocktail,” reflecting the progression of ESC decidualization. Decidualized ESCs are known to dedifferentiate upon removal of the induction media *in vitro*. Indeed, we observed slight decrease in the fluorescent signal 4 days after the media replacement (Figure 3B). Moreover, fluorescence raised again in response to repeated hormonal supplementation (Figure 3B). Of note, undifferentiated cells displayed stably low fluorescence level during the whole observation period. The results of fluorescence estimation by FACS completely coincided with the expression dynamics of the key decidual genes *PRL, IGFBP1, FOXO1*, and *CLU* obtained by RT-PCR (Figure 3C). Together, these results demonstrate that our reporter system reflects decidualization with a high degree of sensitivity.

We next examined the specificity of the developed tool. To do so, along with the five primary ESC lines, we transduced human diploid fibroblasts (HDF) and adipose-derived human mesenchymal stem cells (adMSCs) with the dec_pPRL-mCherry expression vector. As expected, in both cell lines of non-endometrial origin, there was almost no increase in mCherry fluorescence as well as no upregulation in the expression of the most important decidual marker genes *PRL* and *IGFBP1* upon hormonal treatment (Figures 3D,E, Supplementary Figure 1A). On the contrary, all ESC lines reacted significantly to the induction media as indicated by more than 10-fold increase in fluorescence (Figures 3D,E). Together, these suggest that cell types of non-endometrial origin lack the appropriate transcription factor repertoire to utilize MER20/MER39 cassette as a regulatory platform to drive mCherry expression. The absence of the appropriate TF repertoire in cells of nonendometrial origin was additionally verified by the qPCR for *PGR, ESR1, XBP1*, and *CEBP/B* (Supplementary Figure 1B). Finally, we have developed rather sensitive and specific tool to estimate decidual reaction of ESCs.

### Dec_pPRL-Mcherry Tool Can Be Applied for the Personalized Selection of the Hormonal Supplementation Scheme

**Figure 4:**
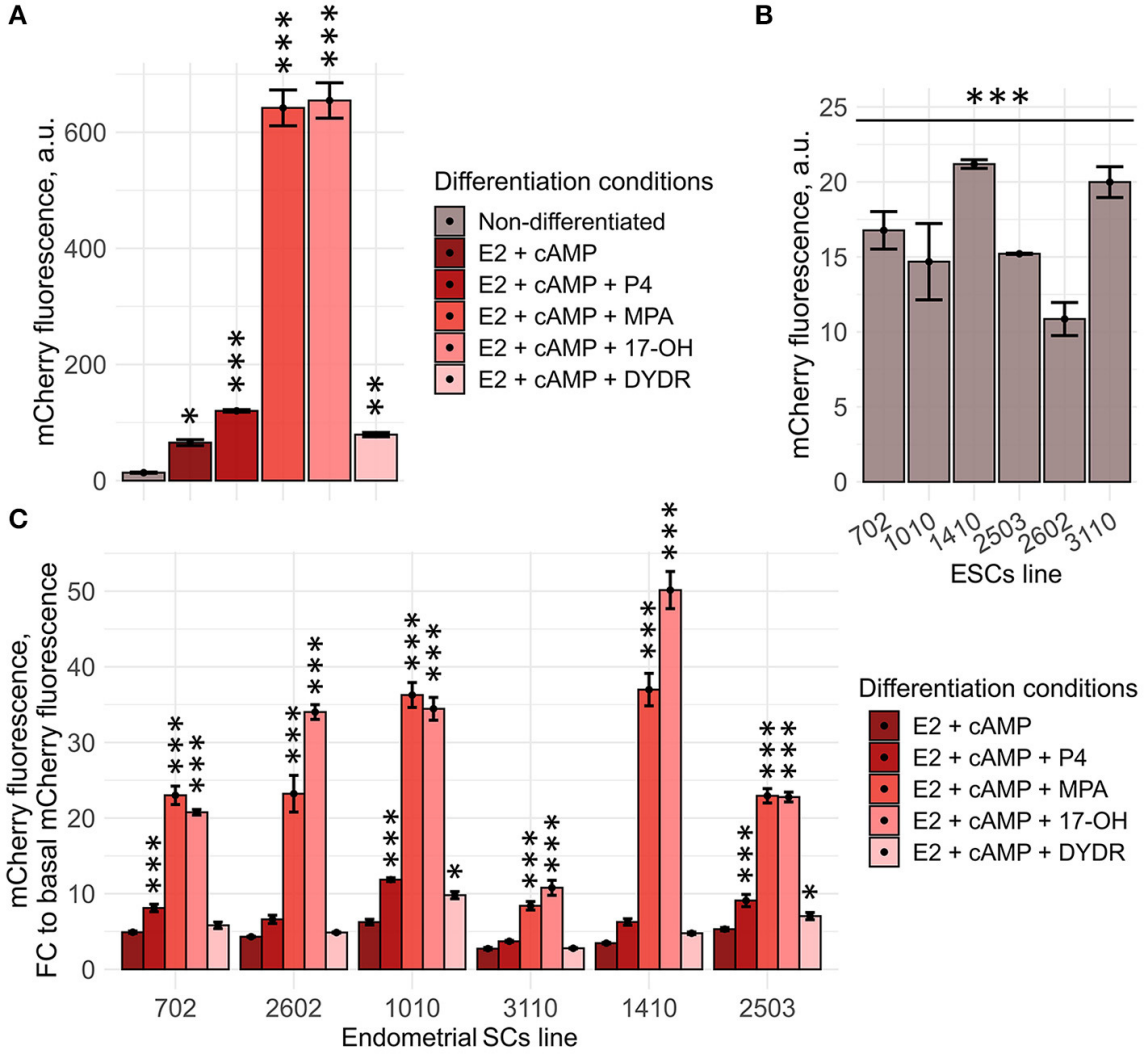
Generated Dec_pPRL-mCherry tool can be applied for personalized screening of the sex steroid hormones. **(A)** ESC (line 2804) decidual reaction significantly varies dependently on the hormonal combination applied as indicated by the level of mCherry fluorescence assessed by FACS. Values are M ± S.D. (*N* = 3). *p < 0.05; **p < 0.01; ***p < 0.005 by ANOVA with Tukey HSD vs. non-differetiated cells. A.u., arbitrary units. **(B)** Various ESC lines differ in the basal mCherry fluorescence levels. Values are M ± S.D. (*N* = 3). ***p < 0.005 by one-way ANOVA. A.u., arbitrary units. **(C)** Tested ESC lines responded differently toward various “hormonal cocktails” applied to induce decidualization. Values are presented as fold changes reflecting the ratios of mCherry fluorescence on day 8 of differentiation to the appropriate basal fluorescence, calculated for each ESC line and compound combination tested. M ± S.D. (*N* = 3). *p < 0.05; ***p < 0.005 by ANOVA with Tukey HSD vs. (E2 + cAMP)-treated ESCs.

One of the feasible applications of the designed system seemed to be the selection of the patient-specific hormonal supplementation scheme. To test this suggestion, we initially compared decidual reaction of the primary ESC line expressing Dec_pPRL-mCherry vector toward progesterone (P4) and synthetic progestogens, including MPA, dydrogesterone (DYDR), or 17-hydroxyprogesterone caproate (17-OH). To unify the experimental conditions, E2 and cAMP were added to each group tested, except for the untreated one. As shown in Figure 4A, decidual response varied significantly depending on the type of progestogen applied. The most abundant reaction was achieved when ESCs were treated with MPA or 17-OH, whereas the lowest response was induced by DYDR (Figure 4A). Thus, our genetic tool is sensitive enough to compare stromal reaction induced by various hormonal analogs and may be used to choose the most appropriate one.

In order to extend our observations, we further reproduced the above experimental approach using six ESC lines obtained from patients who were planning to undergo the IVF procedure. It should be specifically highlighted that both selection of the suitable donor and biomaterial sampling procedures were strictly regulated, nevertheless, isolated ESC lines varied to some extent in the basal mCherry fluorescence levels [though the viral integration frequencies were comparable between all the tested lines (Supplementary Figure 2)] (Figure 4B). Of note, this difference should be necessarily taken into account, since it could significantly distort the results of the comparison. To avoid any possible errors in the further analysis, we normalized mCherry fluorescence induced by either stimulus per basal mCherry fluorescence for each cell line tested. Obtained fold changes were then used for the comparison. In order to verify the results obtained using Dec_pPRL-mCherry vector, we assessed expression levels of the key decidual marker genes (*PRL* and *IGFBP1*) as well as the described TFs for two ESC lines (Supplementary Figures 3A,B). Based on the data presented in Figure 4C, Supplementary Figure 3, several important conclusions logically flowed out. Firstly, there was a clear patient-specific difference in the ESC decidual reaction, what might reflect variations in the receptivity of endometrial stroma. Secondly, supplementation with the diverse progesterone analogs enhanced decidualization of different ESC lines to varying degrees, demonstrating the necessity for personalized hormonal selection. For example, in case of the 3110 ESC lines that turned out to be the most insensitive toward hormonal treatment, there would be no benefit in using P4 or DYDR, as these compounds did not display any additional inducing effect, while application of 17-OH led to an obvious increase in decidual response and thus might have some positive impact on endometrial receptivity (Figure 4C). Furthermore, we tested whether increasing progestagens' concentrations can enhance decidual response of ESCs. To do so, we estimated mCherry fluorescence of the low-responsive cell line 3,110 toward increasing concentrations of various progestagens (1, 5, and 15 μM). Indeed, increasing concentrations to 5 μM led to a more pronounced decidual response for all the tested compounds (Supplementary Figure 4A). However, P4 and DYDR even at concentrations of 5 μM were not able to induce more pronounced decidualization compared with 1 μM of MPA or 17-OH. Interestingly, increasing concentration to 15 μM did not further enhance decidual reaction of ESC line 3,110, except for DYDR (Supplementary Figure 4A). Moreover, such an increase in concentration resulted in cell death as indicated in Supplementary Figure 4B. These results correlate well with the recently published data showing that the high concentration of progesterone is harmful for endometrial receptivity and decidualization (Liang et al., 2018).

Finally, we can conclude that the proposed approach comprising the genetic reporter system based on the activation of the MER20 and MER39-containing decidual *PRL* promoter, on the one hand, and the model of *in vitro* ESC decidualization, on the other hand, represents a novel tool to assess endometrial receptivity for personalized screening of female sex steroid hormones.

## Discussion

Since IVF became routinely applied for infertility treatment, huge efforts are spent to increase its effectiveness. Currently, this issue has become particularly acute as the trend of the last 4 years indicates a significant decline in the positive clinical outcomes of IVF (Gleicher et al., 2019). The observed decrease in the live birth rates is partially due to the obvious patients' desire to increase the safety of the IVF procedure for the maternal organism and to obtain the predictable outcome (reduce the rate of multiple pregnancies) (Martikainen et al., 2001). This point is satisfied by the elective single embryo transfer, what, however, heavily influences IVF success rates (Gleicher et al., 2019). Such a correlation between the use of a single embryo and the decline in the IVF efficacy dictates a certain need for personalization of the applied approaches. Indeed, today, there is a tendency toward the development of the high-tech patient-specific strategies in IVF; for example, preimplantation genetic testing for aneuploidy (PGT-A) using NGS technologies gradually becomes an integral part during IVF (Brezina et al., 2016). PGT-A test allows selecting the best embryo for the transfer by analyzing genetic background of the separate blastomers composing embryo. Less-risky variation of the PGT-A also available today is non-invasive PGT-A based on the sequencing cell-free DNA released into the culture medium from both trophectoderm and inner cell mass (Huang et al., 2019). Other brilliant examples of personalized tools using the latest scientific technologies for IVF improvement are endometrial reciptivity analysis (ERA) and window implantation (WIN) tests (Haouzi et al., 2009; Díaz-Gimeno et al., 2011; Messaoudi et al., 2019). The fundamental basis of these techniques is a shift in gene expression profile associated with the transition of the human endometrium from a prereceptive to a receptive state. Both tests involve multiple gene expression analyses combined with the appropriate bioinformatics to define short period of time, when endometrium is properly developed to accept transferring embryo.

Within the present study, we also focused on the adequate endometrium preparation as the strategy to increase success of IVF. To this end, we designed the patient-specific genetic tool to optimize hormonal supplementation schemes required for the maintenance of endometrial receptivity during luteal phase. Luteal phase support seems to be a live issue, as progesterone insufficiency to maintain a regulatory secretory endometrium is a fairly common consequence of hormonal stimulation in IVF protocols (Van der Linden et al., 2011; Palomba et al., 2015). Thereby, today, a lot of studies are held to select the suitable dosage, duration, and administration routes for progesterone that is typically applied for luteal phase support (Palomba et al., 2015; Watters et al., 2020). Moreover, much attention is paid to choose more effective alternatives to natural progesterone by testing various progestins and/or other hormonal combinations for endometrial receptivity maintenance during luteal phase (Abate et al., 1999; Yu et al., 2018; Brum Scheffer et al., 2019; Fusi et al., 2019; Griesinger et al., 2019). Importantly, most of the conclusions relative to this problem are obtained *via* randomized clinical trials (RCTs) and corresponding systematic reviews, retrospective observational studies, and meta-analysis. In order to convert observations of RCTs to reliable findings, such trials should be performed on the large cohorts. Moreover, clinical studies may have unpredictable side effects for the participants. Limitations of the systematic reviews and meta-analysis are always inherent to the limitations of the included RCTs. Even more important that such approaches are far from being patient specific, on the contrary, they imply certain level of unification both of the patients and the provided recommendations.

The starting point to generate genetic tool assessing endometrial receptivity was selection of the appropriate testing model, and *in vitro* decidualization of ESCs perfectly matched our selection criteria. Being the physiological reaction of the endometrial stroma toward the ovarian steroids, decidualization reflects the transition of the endometrium to a receptive state and is a prerequisite for successful blastocyst implantation (Okada et al., 2018). Today, the vast majority of fundamental studies unraveling various aspects of endometrial receptivity, such as identification of the key molecular regulators and participants, interactions with trophoblast or uterine NK cells, are performed using ESC *in vitro* deidualization model (Gellersen et al., 2010; Garrido-Gomez et al., 2017; Lucas et al., 2020). Though slightly different cocktails are used to induce decidualization of ESCs, most of them are combinations of progesterone/progestin, E2, and cAMP (reviewed in Gellersen and Brosens, 2014). Here, we applied the most common induction mixture containing all three agents. Treating primary ESCs with this cocktail led to the significant alterations in gene expression profile, differentially expressed genes clustered in processes relevant to the decidualization, including extracellular matrix remodeling and regulation of the local immune response. As expected, we observed the appearance of the classic decidual markers—*PRL* and *IGFBP1*. Thereby, *in vitro* decidualization of ESCs may be used as the model to estimate endometrial receptivity.

The preferable features of the diagnostic tools to become applicable in the routine clinical testing are the simplicity in performance together with the representativeness regarding the assessed parameter. In this context, the integral characteristic reflecting the activity of the whole decidual network seems to be more appropriate than estimation of the expression levels of a predefined set of genes involved in ESC decidualization. The fundamental background suggesting the existence of the integral molecular regulators that orchestrate ESC decidual reaction can be found in the phylogenetic studies on the evolution of pregnancy in Eutherian mammals (Lynch et al., 2011, 2015; Emera and Wagner, 2012; Emera et al., 2012). It is known that ESC decidualization evolved exactly in Eutherians and, therefore, is considered to underlie the key distinctive features of the prolonged pregnancy in Eutherian mammals, including direct implantation of the blastocyst into maternal endometrium, feto-maternal communication, and maternal immunotolerance to semi-allogenic fetus (Emera et al., 2012). When tracing the molecular origin of the regulatory landscape that manages decidualization, crucial role for the ancient mammalian transposable elements in orchestrating the transcriptional response of decidualizing ESCs was established (Emera and Wagner, 2012; Emera et al., 2012). Among the described ancient transposons involved in the regulation of the decidual gene network, MER20 and MER39 seem to be the most characterized (Gerlo et al., 2006; Lynch et al., 2011; Emera and Wagner, 2012; Gellersen and Brosens, 2014). Both transposons are inserted into the decidual PRL promoter, thus controlling *PRL* expression in differentiated ESCs (Emera and Wagner, 2012). Moreover, in previous studies, MER20 was found within the 200-kb window around the TSS of the other genes differentially regulated upon decidualization, suggesting that ESCs utilize MER20 as progesterone/cAMP-responsive elements to drive expression of decidual-specific genes (Lynch et al., 2011). In line with the other authors, we observed that the sequence of MER20/MER39 transposons donated binding motifs for the core decidual transcription factors CEBP/B, PR, STAT5A, XBP-1, and ER (Lynch et al., 2011). Moreover, we revealed that these transcription factors were upregulated and controlled activity of the vast majority of the differentially expressed genes in decidual ESCs. Together, this led us to the suggestion that MER20/MER39 cassette may function as a platform for the key transcription factors that regulate ESC decidualization, and its activation may reflect the adequacy of the transcription regulators profile that manages the whole decidual program in ESCs. Thus, the short sequence of *dPRL* promoter containing MER20 and MER39 can be utilized to estimate endometrial receptivity.

Having established the “seed” element of the designed genetic tool for endometrial receptivity assessment, the next step was to develop the simple approach to evaluate its activity. To this end, using molecular cloning, we constructed lentivector-containing fluorescent reporter controlled by the decidual PRL promoter including MER20 and part of MER39 sequences. Thus, endometrial receptivity could be easily estimated by the fluorescent intensity of transduced ESCs upon decidualization stimuli. As expected, fluorescent level correlated well with the expression of the core decidual genes, namely *PRL, IGFBP1, FOXO1*, and *CLU*. It should be specifically highlighted that neither adMSCs nor HDF transduced with the constructed lentivector, thus carrying progesterone/cAMP-responsive elements within MER20 and MER39 sequences inserted into the genome, displayed significant increase in fluorescence in response to exogenous hormones and cAMP composing decidualization cocktail. The latter demonstrates specificity of our genetic tool and proves the lack of the appropriate transcription factor repertoire to utilize MER20/MER39 as progesterone/cAMP-responsive regulatory elements in cell types other than ESCs.

Interestingly, almost simultaneously with our research, another group developed a high-throughput screening tool to study the molecular regulators of decidualization at the genetic level (Haller et al., 2019). Specifically, the authors used immortalized human ESC line transduced with the genetic construct that upon recombination carried yellow fluorescent protein under the PRL promoter. This system was used to perform a genome-wide siRNA library screen and thus to reveal genetic and chemical modulators of decidualization. Though we were not able to find the exact parameters and characteristics of the cloned PRL promoter region, the authors also suggest that the developed construct based on the activity of the PRL promoter reflects the quantifiable visual readout of the overall decidualization response.

**Figure 5:**
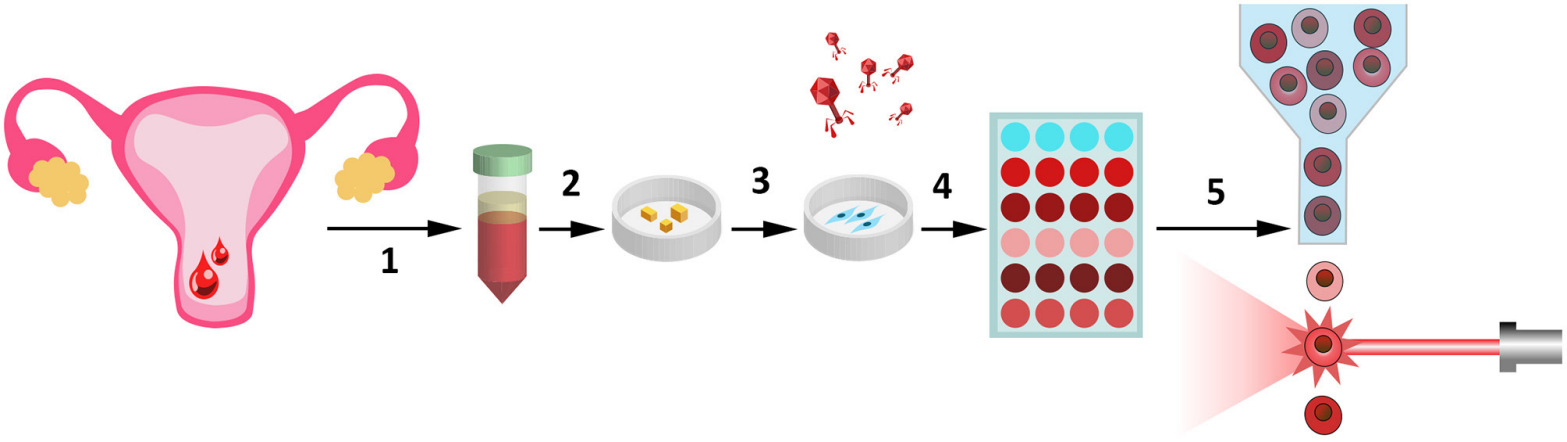
Possible clinical adaptation of the designed genetic tool assessing endometrial receptivity for personalized screening of female sex steroid hormones. Several stages of the clinical adaptation of our tool are presented here. (1) To receive the biological samples of endometrial tissue. This non-invasive and non-traumatic procedure can be realized simply by collection of the desquamated endometrium, contained in menstrual blood (Zemelko et al., 2012; Bozorgmehr et al., 2020). (2) Pieces of endometrium are plated on the Petri dishes and (3) patient-specific ESC lines can be easily obtained due to adhesive properties of these cells. (4) Transduction of the primary ESCs with the lentiviruses encoding our genetic construct (Deryabin et al., 2019). (5) Decidual response of ESCs toward various hormonal combinations can be compared by flow cytometry analysis.

Today, progesterone, 17-OH, and DYDR are clinically approved for luteal phase support (Griesinger et al., 2019). In fact, it remains unclear on what basis one or another compound is preferred. Often the selection of the drug is due to the ease of application (orally vs. other routes). While progesterone can be administered orally, intramuscularly, vaginally, or rectally, DYDR is administered orally due to suggested enhanced oral bioavailability (Schindler et al., 2003). Therefore, DYDR seems to be prescribed more often. 17-OH was shown to be more effective for luteal phase support compared with progesterone (Unfer et al., 2004) though it is rather rarely prescribed. Bearing in mind the absence of the concrete strategy for drug prescription during luteal phase support, our tool can provide solid base to select the appropriate substance. Possible clinical adaptation of our genetic tool is displayed in Figure 5. By applying this protocol, we were able to compare the responsiveness of several ESC lines toward different progesterone analogs. Of note, ESC lines differed significantly from one another in the decidual response caused by either combination. Nevertheless, 17-OH and MPA turned out to be the most effective for all the tested lines. In favor of this observation, 17-OH was shown to be the drug of choice in the support of the luteal phase (Abate et al., 1999). However, going back to the patient-specificity, in one of the tested ESC lines, 17-OH caused an ~15 times rise in the fluorescence level, suggesting an extremely intense decidual reaction. Such a hyper-reaction of endometrium may be somewhat deleterious, as rapid or early secretory transformation and/or abnormal endometrial development can impair optimal uterine receptivity and embryo implantation by disrupting embryo-endometrial synchrony (Teh et al., 2016). Recently, it was shown that premature expression of the decidualization marker PRL during the luteal phase is associated with recurrent implantation failure (Berkhout et al., 2020). In hyper-reactive cells, decidual reaction is equally likely to be more pronounced or develop more rapidly peaking earlier than it is supposed to, both leading to inappropriate expression and secretion profile at the moment of implantation. Therefore, choosing another compound for this specific patient might be more preferable. On the contrary, among the lines tested, there was the very low-responsive one. Hypo-reaction revealed using our genetic tool allows suggesting that such ESCs lack appropriate expression profile, thus their decidualization is impaired. The correlation between impaired decidualization and implantation failures has a lot of evidence in the literature. For example, ESCs purified from women suffering from recurrent pregnancy loss were characterized by decreased decidualization (particularly by decreased PRL expression) (Salker et al., 2010). In case of this patient, supplementation with natural progesterone or dydrogesterone would not provide any additional stimulating effect, whereas 17-OH could cause enhancement of the decidual response. Unexpected results were obtained for dydrogesterone. This progestin is almost as often prescribed as the progesterone itself to support the luteal phase (Barbosa et al., 2016; Griesinger et al., 2018, 2019). Moreover, it was suggested that dydrogesterone may be even more preferable for this purpose than natural progesterone (Griesinger et al., 2019). According to our results, dydrogesterone caused the modest decidual reaction compared with all the tested compounds in each ESC line. One of the possible strategies to enhance decidualization of the low-responsive ESCs might seem to be the increase in the concentration of the drug applied for luteal phase support. Indeed, according to our data, slight increase in the concentration of progestagens may enhance ESC decidualization to some extent; however, further rise in the concentration did not intensify decidual response while inducing ESC death. These results are in line with the recently published data demonstrating that excess of progesterone compromises *in vitro* decidualization of ESCs and negatively affects embryo implantation in the mouse models (Liang et al., 2018).

To sum up, the all-in-one genetic tool based on the MER20/MER39 expression cassette provides the ability to predict the most appropriate hormonal cocktail for endometrial receptivity maintenance specifically and safely for the patient, and thus to define the personal treatment strategy prior to IVF procedure.

## Data Availability Statement

The datasets presented in this study can be found in the GEO database under accession number GSE160702.

## Ethics Statement

The studies involving human participants were reviewed and approved by The Local Bioethics Committee of the Institute of Cytology of the Russian Academy. The patients/participants provided their written informed consent to participate in this study.

## Author Contributions

AB supervised the work and wrote and edited the manuscript. AB and PD designed the experiments and conducted the studies. PD performed statistical and bioinformatic analysis of the obtained data and molecular cloning experiments and edited the manuscript. IG, MR, and MP obtained patients' materials. AD isolated primary ESC lines from donor samples. NN assisted in manuscript editing. All authors contributed to the article and approved the submitted version.

## Conflict of Interest

The authors declare that the research was conducted in the absence of any commercial or financial relationships that could be construed as a potential conflict of interest.

## Supplementary Material

Supplementary Figure 1 Expression of the **(A)** key decidual marker genes *PRL* and *IGFBP1* as well as **(B)** the TFs regulating decidualization in ESCs, HDF, and Ad-MSCs by RT-PCR. Values are M ± S.D. (*N* = 3). ^*^*p* < 0.05; ^**^*p* < 0.01; ^***^*p* < 0.005 differentiated vs. non-differentiated cells by Student's *t*-test.

Supplementary Figure 2 Viral integration frequencies assessed for the ESC lines. Viral integration frequency was assessed according to the procedure described by Barczak et al. (2015). In brief, lentiviral copy number at an integrated lentiviral DNA level was estimated as the relation of exogenous Woodchuck Hepatitis Virus Posttranscriptional Regulatory Element (WPRE) to endogenous single-copy albumin gene by real-time PCR. Values are M ± S.D. (*N* = 3).

Supplementary Figure 3 Expression of the **(A)** key decidual marker genes *PRL* and *IGFBP1* as well as **(B)** the TFs regulating decidualization in 2503 and 3110 primary ESC lines. Decidualization was induced by E2+cAMP+MPA and assessed on day 8 of differentiation. Values are M ± S.D. (*N* = 3). ^*^*p* < 0.05; ^**^*p* < 0.01; and ^***^*p* < 0.005 differentiated vs. non-differentiated cells by Student's *t*-test.

Supplementary Figure 4 Effects of the increasing doses of progestagens on **(A)** decidualization of ESCs (line 3110) assessed using Dec_pPRL-mCherry reporter system and **(B)** the corresponding cell viability. *x*-Axis in the dot plots is the intensity of mCherry fluorescence reflecting decidualization progression. *y*-Axis is the intensity of DAPI fluorescence reflecting dying cells. Values are M ± S.D. (*N* = 3). ^*^*p* < 0.05; ^**^*p* < 0.01, ^***^*p* < 0.005 by ANOVA with Tukey HSD vs. standard 1 μM progesatgen concentration for each compound.

Supplementary Table 1 The results of DE analysis of decidualized vs. non-differentiated ESCs.

Supplementary Table 2 Subset of DEGs identified from DE analysis of decidualized vs. non-differentiated ESCs.

Supplementary Table 3 Gene set enrichment analysis for biological processes and pathways in KEGG pathway DB of decidualized vs. non-differentiated ESCs.

Supplementary Table 4 Gene set enrichment analysis in terms of regulation by TFs based on TRED DB built on the preranked gene list by absolute value of LFC from DE analysis of decidualized vs. non-differentiated ESCs.

Supplementary Table 5 Gene set enrichment analysis in terms of regulation by TFs based on TRED DB build on the pre-ranked gene list by LFC from DE analysis of decidualized vs. non-differentiated ESCs.

## Abbreviations

cAMP: cyclic adenosine monophosphate
DEGs: differentially expressed genes
*dPRL*: decidual *PRL* promoter
DYDR: dydrogesterone
E2: β-estradiol
ESCs: endometrial stromal cells
ER: estrogen receptor
ESR1: estrogen receptor gene
IVF: *in vitro* fertilization
LFC: log fold change
MPA: medroxyprogesterone 17-acetate
P4: progesterone
PRL: prolactin
PR: progesterone receptor
PGR: progesterone receptor gene
TFs: transcription factors
TSS: transcription start site
17-OH: 17α-hydroxyprogesterone caproate.
