# Supplementary figures and images for: “All-In-One” Genetic Tool Assessing Endometrial Receptivity for Personalized Screening of Female Sex Steroid Hormones
Source: Front Cell Dev Biol. 2021 Feb 15;9:624053. doi: 10.3389/fcell.2021.624053 (PMC7917288; doi:10.3389/fcell.2021.624053)

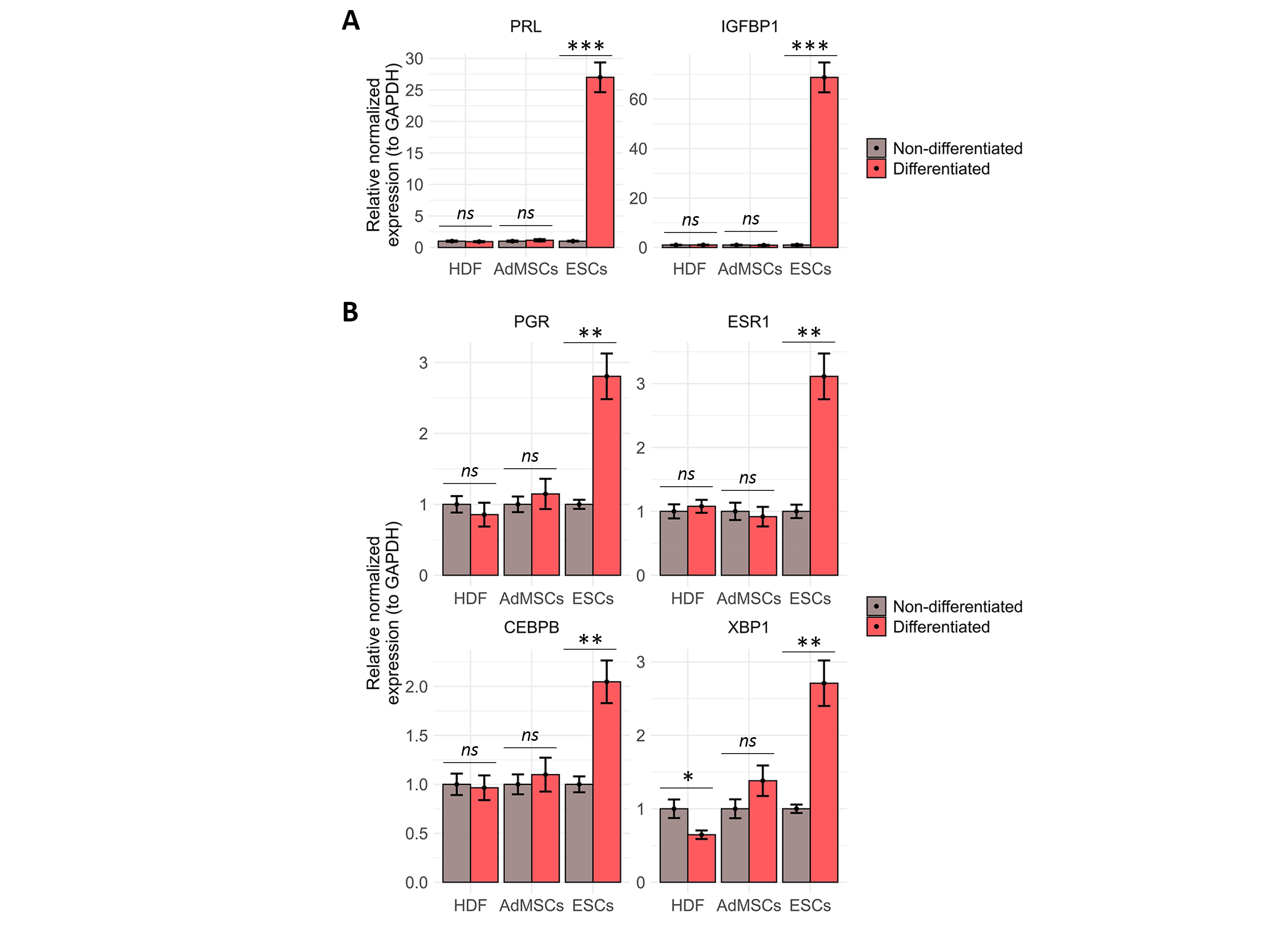

Supplement: Supplementary Figure 1 — Expression of the (A) key decidual marker genes PRL and IGFBP1 as well as (B) the TFs regulating decidualization in ESCs, HDF, and Ad-MSCs by RT-PCR. Values are M ± S.D. (N = 3). *p < 0.05; **p < 0.01; ***p < 0.005 differentiated vs. non-differentiated cells by Student's t-test. [file Image_1.tif]

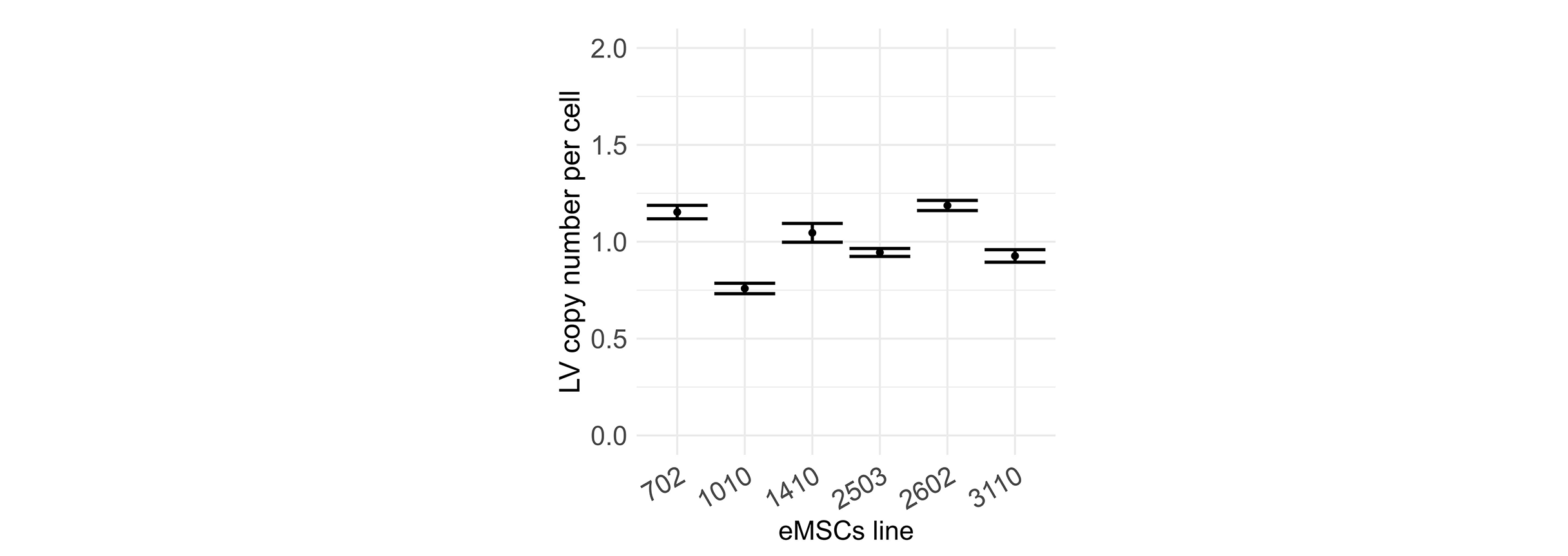

Supplement: Supplementary Figure 2 — Viral integration frequencies assessed for the ESC lines. Viral integration frequency was assessed according to the procedure described by Barczak et al. (2015). In brief, lentiviral copy number at an integrated lentiviral DNA level was estimated as the relation of exogenous Woodchuck Hepatitis Virus Posttranscriptional Regulatory Element (WPRE) to endogenous single-copy albumin gene by real-time PCR. Values are M ± S.D. (N = 3). [file Image_2.tif]

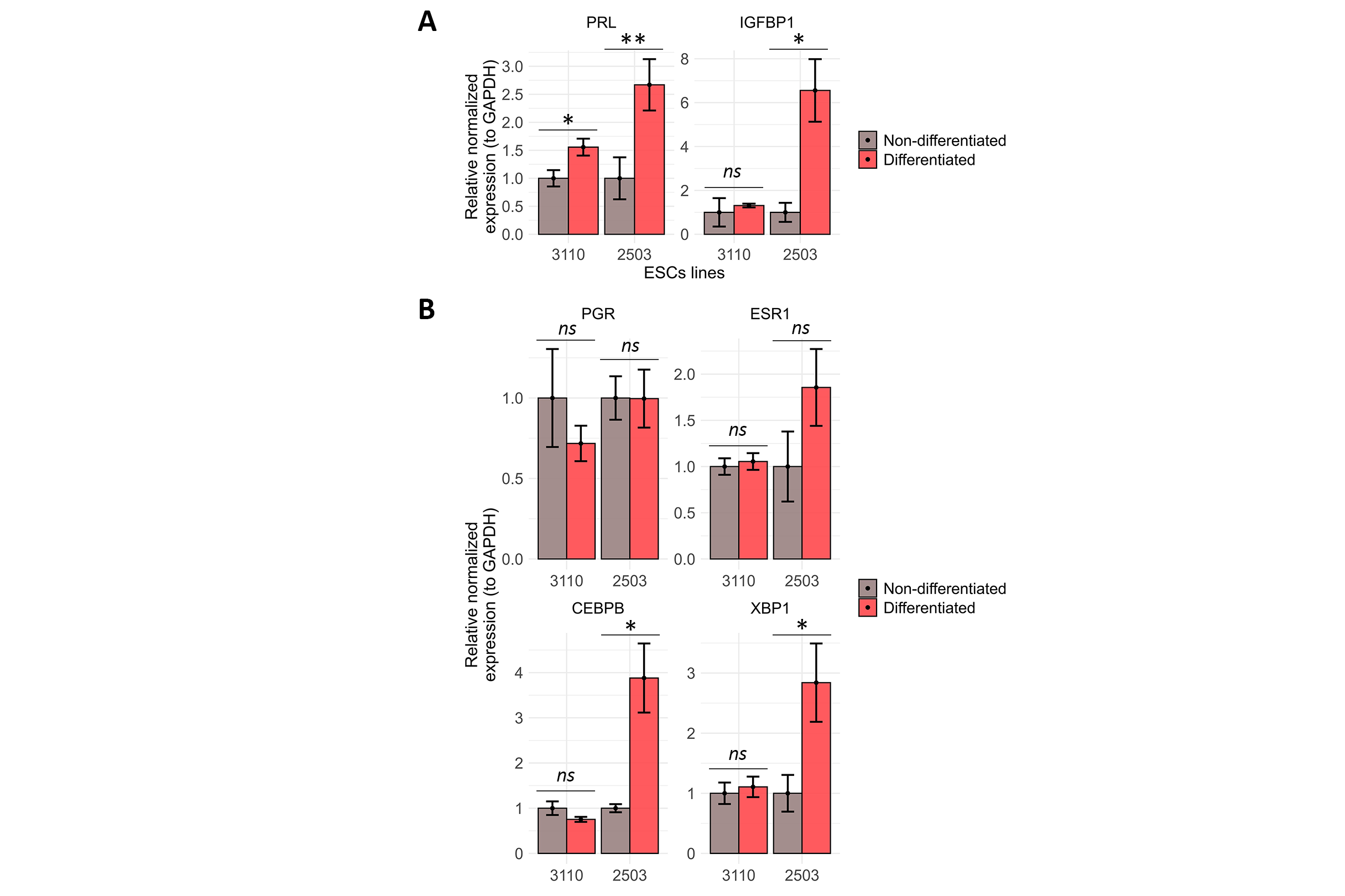

Supplement: Supplementary Figure 3 — Expression of the (A) key decidual marker genes PRL and IGFBP1 as well as (B) the TFs regulating decidualization in 2503 and 3110 primary ESC lines. Decidualization was induced by E2+cAMP+MPA and assessed on day 8 of differentiation. Values are M ± S.D. (N = 3). *p < 0.05; **p < 0.01; and ***p < 0.005 differentiated vs. non-differentiated cells by Student's t-test. [file Image_3.tif]

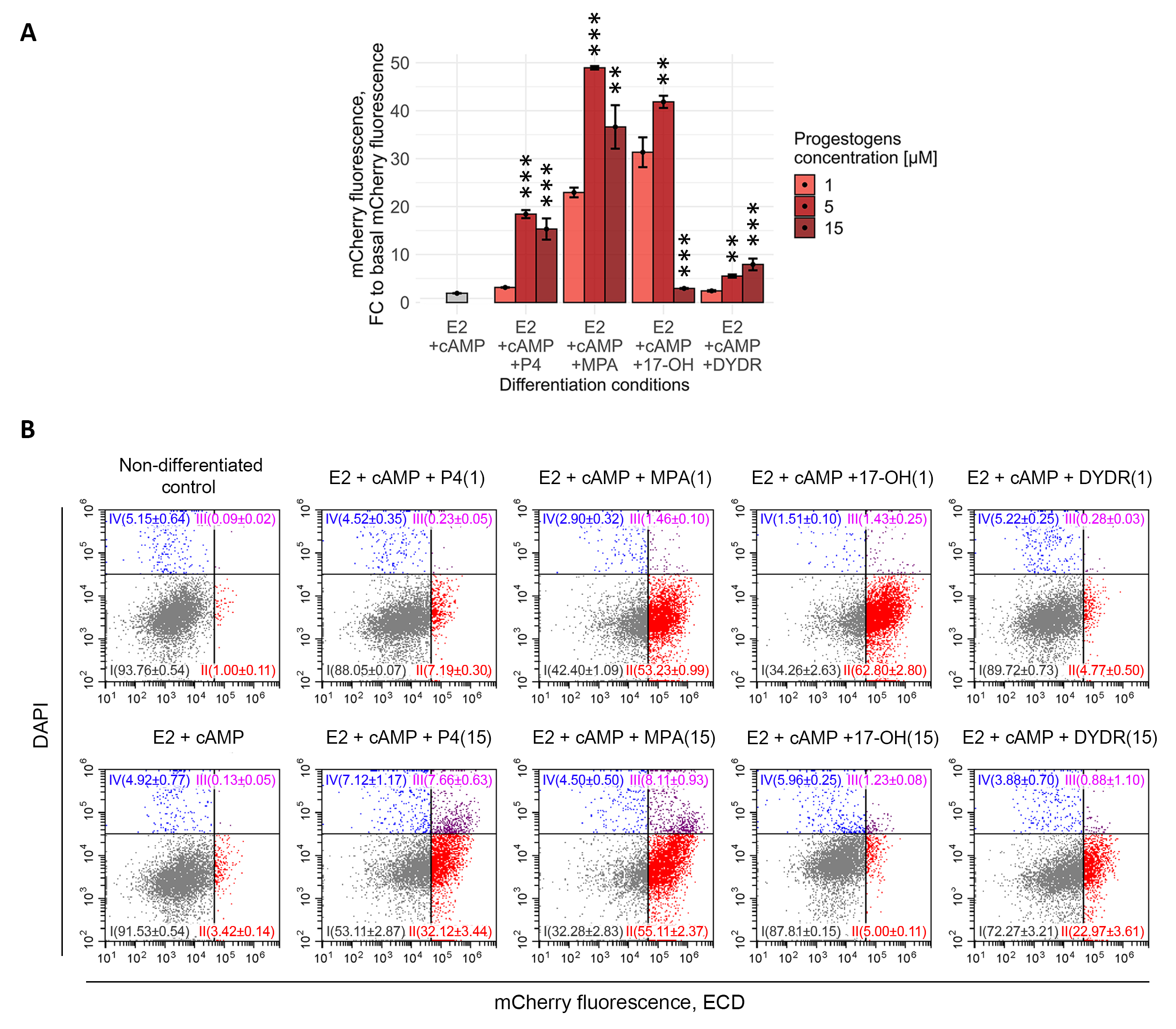

Supplement: Supplementary Figure 4 — Effects of the increasing doses of progestagens on (A) decidualization of ESCs (line 3110) assessed using Dec_pPRL-mCherry reporter system and (B) the corresponding cell viability. x-Axis in the dot plots is the intensity of mCherry fluorescence reflecting decidualization progression. y-Axis is the intensity of DAPI fluorescence reflecting dying cells. Values are M ± S.D. (N = 3). *p < 0.05; **p < 0.01, ***p < 0.005 by ANOVA with Tukey HSD vs. standard 1 μM progesatgen concentration for each compound. [file Image_4.tif]
